# Neurotrophic delivery in Alzheimer’s-model mice

**DOI:** 10.1186/2050-5736-3-S1-O18

**Published:** 2015-06-30

**Authors:** Elisa Konofagou, Hong Chen, Oluyemi Olumolade, Karen Duff

**Affiliations:** 1 Columbia University, New York, NY, United States

## Background/introduction

Alzheimer’s disease (AD), which has emerged as one of the most common brain disorders, begins in the hippocampal formation and gradually spreads to the remaining brain at its most advanced stages, and is characterized partly by deposition of amyloid plaques in the brain tissue but also in the blood vessels themselves. Our studies have dealt with both the delivery of neurotrophic factors through the FUS-induced blood-brain barrier (BBB) opening to the hippocampus in both the presence and absence of disease in AD mouse models. The Brain-Derived Neurotrophic Factor (BDNF) is widely and abundantly expressed in the CNS and is available to some peripheral nervous system neurons that uptake the neurotrophin produced by peripheral tissues. BDNF can modulate neuronal synaptic strength and has been implicated in hippocampal mechanisms of learning and memory. As BDNF has been proven to serve as a neuroprotective agent, delivery of exogenous BDNF is a good candidate for therapeutic treatment of several CNS disorders.

## Methods

FUS at 1.5 MHz with in-house microbubbles of an average diameter of 4-5 mm was performed in transgenic AD mice. Two transgenic mouse models, the PS/APP and J20, were used, which are characterized by amyloid plaques and cognitive deficits, respectively.

BDNF was injected following BBB opening. The BDNF bioactivity was assessed quantitatively by immunohistochemical detection of the pTrkB receptor and activated pAkt, pMAPK, and pCREB in the hippocampal neurons.

## Results and conclusions

BBB opening using FUS and microbubbles was found to have similar characteristics and follows similar timelines in AD mouse models as in wildtype mice. BDNF was found to trigger molecular pathways with the pTrkB, pMAPK and pCREB showing significant enhancement compared to the contralateral hemisphere (no FUS).

Current efforts include demonstration of therapeutic efficacy of BDNF delivery in AD mouse models. Targeting of the hippocampus in non-human primates will also be shown.

